# Impact of glucocorticoid administration on therapeutic outcomes of immune checkpoint inhibitors in non-small cell lung cancer: a systematic review and meta-analysis

**DOI:** 10.3389/fmed.2025.1649353

**Published:** 2025-10-20

**Authors:** Song Zhang, Shen-Da Chen, Ling Chen, Bo Hong

**Affiliations:** Department of Pulmonary Medicine, Ningbo Municipal Hospital of Traditional Chinese Medicine (TCM), Affiliated Hospital of Zhejiang Chinese Medical University, Ningbo, Zhejiang, China

**Keywords:** non-small cell lung cancer, immune checkpoint inhibitors, glucocorticoids, progression-free survival, overall survival, meta-analysis

## Abstract

**Background:**

Non-small cell lung cancer (NSCLC) remains a leading cause of cancer-related mortality. Immune checkpoint inhibitors (ICIs) have improved outcomes in advanced NSCLC, yet concurrent glucocorticoid use raises concerns due to immunosuppressive effects. Evidence regarding the prognostic impact of glucocorticoids in this setting remains inconsistent. This study aimed to systematically evaluate the association between glucocorticoid use and survival outcomes in NSCLC patients receiving ICIs, with particular attention to timing of administration.

**Methods:**

A systematic search of PubMed, Embase, Web of Science, and the Cochrane Library was conducted up to December 18, 2024, with no language restrictions. Eligible studies enrolled adult NSCLC patients treated with ICIs, stratified by glucocorticoid exposure, and reported overall survival (OS), progression-free survival (PFS), or objective response rate (ORR). Pooled hazard ratios (HRs) with 95% confidence intervals (CIs) were estimated using fixed- or random-effects models depending on heterogeneity. Study quality was appraised with the Newcastle–Ottawa Scale, and evidence certainty was evaluated using GRADE. Subgroup analyses were conducted to assess the impact of glucocorticoid timing (pre-ICI, at initiation, and post-ICI) on survival outcomes.

**Results:**

Fifteen studies involving 5,950 patients were included. Glucocorticoid use was significantly associated with inferior outcomes. The pooled HR for PFS was 1.44 (95% CI, 1.15–1.73; *p* < 0.001; I^2^ = 77.7%), and for OS was 1.58 (95% CI, 1.24–1.93; *p* < 0.001; I^2^ = 84.2%). Subgroup analysis demonstrated that post-ICI glucocorticoid administration was strongly associated with poorer survival (PFS: HR = 1.98, 95% CI, 1.51–2.62; OS: HR = 2.28, 95% CI, 1.61–3.41; both *p* < 0.001), while pre-ICI use showed no significant effect (PFS: HR = 1.21, 95% CI, 0.85–2.01; OS: HR = 1.31, 95% CI, 0.69–2.28). Funnel plots and Egger’s regression test indicated no significant publication bias (PFS: *p* = 0.42; OS: *p* = 0.37). Evidence certainty for both OS and PFS was rated as moderate.

**Conclusion:**

In NSCLC patients receiving ICI therapy, glucocorticoid use might be associated with significantly poorer progression-free and overall survival, particularly when administered after the initiation of ICIs. Further research is warranted to clarify the timing and dosing parameters that could minimize potential negative effects on ICI efficacy.

**Systematic review registration:**

PROSPERO, identifier CRD420251156730, available from https://www.crd.york.ac.uk/PROSPERO/view/CRD420251156730.

## 1 Introduction

Non-small cell lung cancer (NSCLC) remains a leading cause of cancer-related mortality worldwide. Immune checkpoint inhibitors (ICIs) targeting programmed cell death-1 (PD-1), programmed death-ligand 1 (PD-L1), and cytotoxic T-lymphocyte-associated antigen 4 (CTLA-4) have transformed the therapeutic landscape of advanced NSCLC, achieving superior survival benefits compared with conventional chemotherapy. By reinvigorating T-cell-mediated antitumor immunity, ICIs provide durable clinical responses for a subset of patients. Nevertheless, heterogeneity in treatment outcomes persists, and identifying clinical factors that may modify ICI efficacy remains a critical area of investigation (1–3).

Glucocorticoids are frequently used in NSCLC management for diverse indications, including symptom palliation (e.g., cancer-related dyspnea or cachexia), management of brain metastases, treatment of immune-related adverse events (irAEs), and control of comorbid inflammatory conditions. Their potent immunosuppressive effects, however, raise concerns about potential antagonism with ICI-mediated immune activation. By attenuating T-cell proliferation, cytokine release, and antigen presentation, glucocorticoids may diminish the therapeutic efficacy of ICIs (4, 5). Importantly, both the timing and dosage of glucocorticoid administration may be critical modifiers of clinical outcomes. Baseline (pre-ICI) use of corticosteroids, particularly at doses ≥10 mg prednisone-equivalent, has been linked in some studies to inferior survival, possibly reflecting both immunosuppression and confounding by poorer baseline status (6). Conversely, post-ICI corticosteroid use is often related to irAEs, and its prognostic significance may vary according to the severity of toxicity and the intensity of steroid exposure (7, 8).

Despite these concerns, existing evidence on the interaction between glucocorticoid administration and ICI efficacy in NSCLC remains inconclusive. Many studies differ in how they define exposure (timing, dose, and indication), and findings across individual cohorts are inconsistent (9–11). Therefore, a systematic synthesis is needed to clarify whether glucocorticoids modify ICI outcomes and whether the timing of administration differentially affects progression-free survival (PFS) and overall survival (OS). In this systematic review and meta-analysis, we aimed to evaluate the association between glucocorticoid use and ICI outcomes in NSCLC, with specific attention to the timing of exposure (pre-ICI, at ICI initiation, and post-ICI) and the potential implications of dosage.

## 2 Methods

### 2.1 Search strategy

During the systematic review process, we adhered to the PRISMA guidelines (12) (Supplementary Table 1). Four electronic databases (PubMed, Embase, Web of Science, and the Cochrane Library) were searched on December 18, 2024, without applying any time restrictions. The search strategy employed was: ((“Carcinoma, Non-Small-Cell Lung” [Mesh] OR “non-small cell lung cancer” [Title/Abstract] OR NSCLC [Title/Abstract]) AND (“Glucocorticoids” [Mesh] OR glucocorticoid * [Title/Abstract] OR corticosteroid * [Title/Abstract]) AND (“Immune Checkpoint Inhibitors” [Mesh] OR “immune checkpoint inhibitor*” [Title/Abstract] OR “PD-1 inhibitor*” [Title/Abstract] OR “PD-L1 inhibitor*” [Title/Abstract] OR “CTLA-4 inhibitor*” [Title/Abstract])). These keywords were selected based on the PICO framework to ensure a comprehensive retrieval of studies pertinent to the meta-analysis. No language restrictions were applied, and the reference lists of pertinent articles were manually screened for additional records.

### 2.2 Inclusion criteria and exclusion criteria

#### 2.2.1 Inclusion criteria

1) Population: Adult patients (≥18 years) with a histologically or cytologically confirmed diagnosis of non-small cell lung cancer (NSCLC).
2) Intervention/Exposure: Administration of systemic glucocorticoids (e.g., prednisone, prednisolone, dexamethasone, or equivalent) either before or during treatment with immune checkpoint inhibitors (ICIs).
3) Comparator: Patients receiving ICI therapy without concomitant glucocorticoid administration, or comparator groups clearly stratified by glucocorticoid exposure.
4) Outcomes: Studies that reported at least one relevant clinical outcome, including overall survival (OS), progression-free survival (PFS), or objective response rate (ORR), with effect estimates (hazard ratios, odds ratios, or sufficient data to allow their calculation).
5) Study design: Randomized controlled trials (RCTs), prospective cohort studies, retrospective cohort studies, or case–control studies.

#### 2.2.2 Exclusion criteria

1) Study type: Reviews, systematic reviews, meta-analyses, case reports, editorials, commentaries, letters, conference abstracts, or protocols without available original data.
2) Population: Studies that involved mixed cancer populations where NSCLC-specific results could not be extracted separately.
3) Exposure: Studies in which the timing, dosage, or clinical indication for glucocorticoid administration was not clearly defined or could not be ascertained.
4) Outcomes: Studies that did not provide relevant outcomes (OS, PFS, ORR) or did not report sufficient data to calculate effect estimates.
5) Data quality: Duplicate publications or overlapping cohorts. In such cases, the study with the most comprehensive dataset, longest follow-up, or most recent analysis was retained.

### 2.3 Literature screening and data extraction

To ensure transparency and reproducibility, the study selection process was conducted in three stages. First, all records retrieved from the databases were imported into EndNote X9, and duplicate entries were removed. Second, two reviewers independently screened titles and abstracts to exclude irrelevant studies, reviews, case reports, editorials, conference abstracts, and studies lacking original data. Third, the full texts of the remaining articles were assessed against the predefined inclusion and exclusion criteria. Studies were excluded at this stage if they involved mixed cancer types without extractable NSCLC-specific data, failed to provide sufficient information on glucocorticoid timing, dosage, or indication, or presented overlapping patient cohorts. In cases of overlapping reports, the study with the most comprehensive and updated dataset was retained. Any discrepancies were resolved through discussion, with a third reviewer consulted when necessary. The data extracted included the first author, publication year, country, sex ratio, age range, sample size, details of the ICI treatment regimen, details of the glucocorticoid treatment regimen, and outcome measures. When hazard ratios (HRs) and 95% confidence intervals (95%CIs) could not be directly obtained, they were calculated from survival curves using the method described by Tierney et al. (13). Additionally, when the published report did not provide the data of interest, the investigators of the original study were contacted via email to request the unpublished information.

### 2.4 Quality assessment

The quality of the studies included in the meta-analysis was assessed independently by two reviewers using the Newcastle-Ottawa Scale (NOS) (14). This established tool evaluated each study across nine items distributed among three key domains: selection, comparability, and outcome. These domains were employed to identify potential biases inherent in the studies. In the scoring process, one point was assigned for each asterisk noted in the evaluation table, resulting in a total score ranging from 0 to 9. Studies with scores between 0 and 3 were classified as low quality, those with scores from 4 to 6 were considered moderate quality, and studies scoring between 7 and 9 were deemed high quality.

### 2.5 Statistical analyses

Statistical analyses were performed using Stata version 17 (StataCorp, College Station, TX, United States). Initially, heterogeneity among the studies was evaluated using chi-square statistics and quantified by the I^2^ value. When the I^2^ was less than 50% and the corresponding *p*-value was ≥0.10, it was considered that no significant heterogeneity existed, and a fixed-effect model was employed to calculate the pooled effect size. Conversely, when the I^2^ reached or exceeded 50% or the *p*-value was <0.10, significant heterogeneity was inferred, leading to the use of a random-effects model for effect size estimation (15). Subsequently, sensitivity analyses were conducted by sequentially omitting each study to assess the robustness of the overall effect size and to identify potential sources of heterogeneity. Publication bias was assessed using funnel plots and Egger’s regression test. In addition, exploratory meta-regression analyses were conducted using a random-effects model with restricted maximum likelihood (REML) estimation and Knapp–Hartung adjustment to examine whether study-level factors, such as timing of corticosteroid exposure, ICI class, or analytic methodology, contributed to between-study heterogeneity. All statistical tests were two-sided, and a *p*-value <0.05 was regarded as statistically significant.

### 2.6 GRADE assessment of evidence quality

To assess the quality of evidence, the GRADE approach was employed, evaluating key domains: Risk of Bias (study design, blinding, follow-up adequacy); Inconsistency (heterogeneity across studies); Indirectness (relevance to the research question); Imprecision (width of confidence intervals); Publication Bias (symmetry of funnel plots); Large Effect (significant effect sizes); and Dose–Response Gradient (association between dose and effect). Evidence was categorized based on these factors to determine confidence in the effect estimates and guide the interpretation of results.

## 3 Results

### 3.1 Search results and study selection

**Figure 1:**
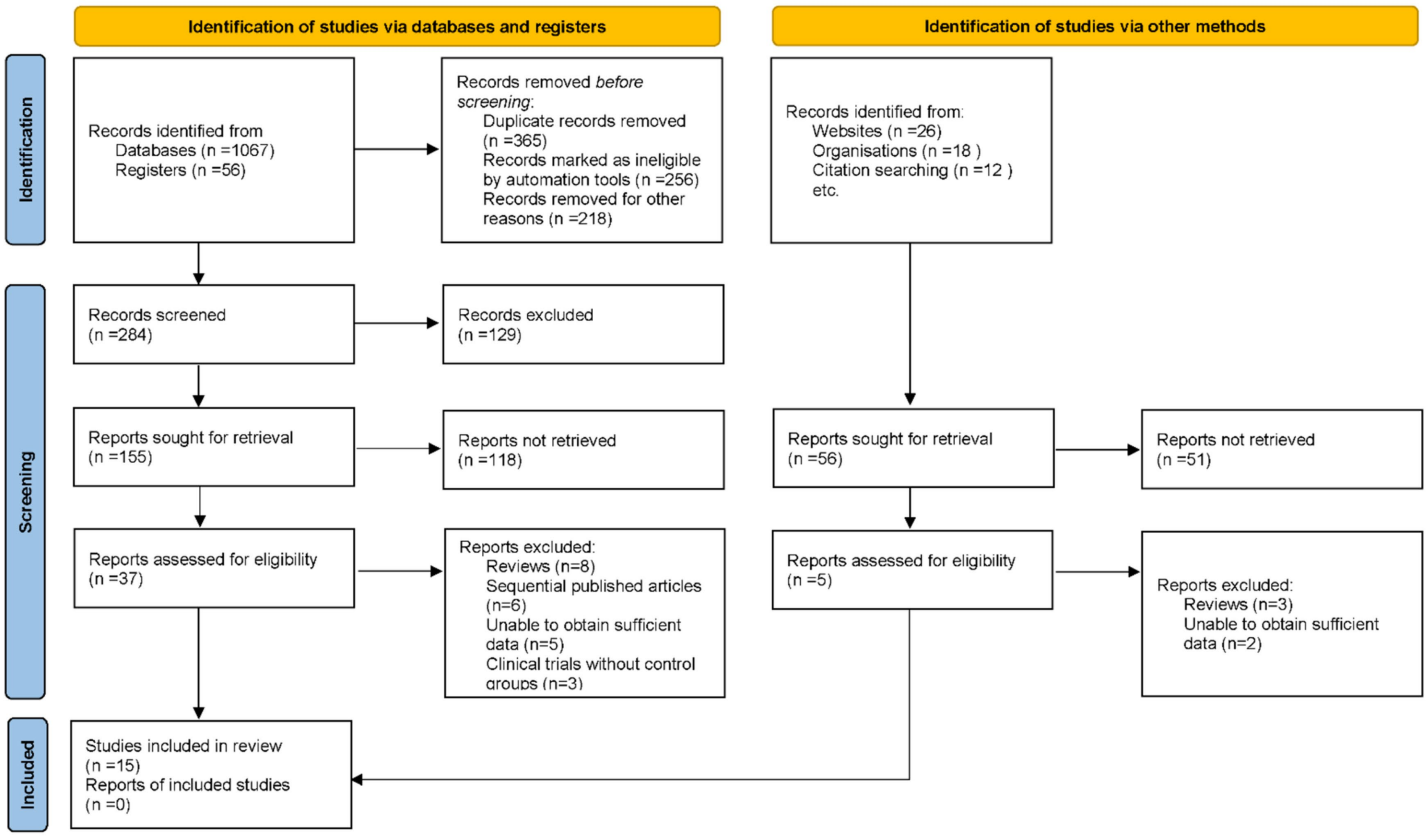
Flowchart illustrating the study selection process for the meta-analysis.

At the inception of this systematic review and meta-analysis, an exhaustive search of multiple electronic databases yielded an initial set of 1,179 potentially relevant articles. Duplicate records were subsequently removed to ensure that each study was uniquely represented. Thereafter, titles and abstracts were screened against pre-established inclusion and exclusion criteria, which encompassed study design, demographic characteristics of the study population, clinical outcomes measured, and overall methodological quality. This preliminary screening resulted in 42 articles being selected for full-text review. Multiple investigators independently assessed the full texts to ensure an unbiased and comprehensive evaluation. During this phase, 27 articles were excluded for the following reasons: review articles (*n* = 11), sequentially published works (*n* = 6), studies with insufficient data for analysis (*n* = 7), and clinical trials lacking control groups (*n* = 3). Ultimately, 15 articles satisfied all the stringent criteria delineated in the research protocol and were included in the final meta-analysis (16–30) (Figure 1).

### 3.2 Study characteristics

A total of fifteen studies were incorporated into the meta-analysis, with individual sample sizes ranging from 67 to 1,025 patients. The median ages across these studies spanned from 63 to 77 years, and the proportion of male participants varied between studies. All studies evaluated the concomitant use of glucocorticoids in patients receiving ICIs for cancer treatment. The ICIs employed were predominantly PD-1/PD-L1 inhibitors, although several studies also included CTLA-4 inhibitors either as monotherapy or in combination with PD-1/PD-L1 agents. Glucocorticoid administration protocols differed considerably among the studies. In some instances, glucocorticoids—primarily prednisone or prednisolone—were administered at the initiation of ICI therapy, whereas other studies reported their use within a 30-day window before or after the commencement of ICI treatment. Dosages varied widely, with some studies reporting relatively low doses (e.g., 6.5 mg) and others employing much higher doses (up to 280 mg or more). Furthermore, the indications for glucocorticoid use were heterogeneous, encompassing management of cancer-related symptoms, brain metastases, palliative care needs, and immune-related adverse events (Table 1).

**Table 1:** Characteristics of studies included in the meta-analysis.

Author (year)	*N*	Male (*n*)	Median age (years)	Glucocorticoid users (*n*)	ICI type	Administration & dosage	Usage indication
Pre-ICI administration							
Cortellini (2021) (18)	950	625	70 (28–92)	228	Pembrolizumab	≥10 mg within 30 days pre-ICI	Not available
Facchinetti (2020) (22)	153	108	<70 (52%) / ≥70 (48%)	47 (prednisone)	Pembrolizumab	At ≥10 mg before ICI initiation	Palliative (100%)
Taniguchi (2017) (29)	201	135	68 (27–87)	23	Nivolumab	6.5 mg (1.56–12.5 mg); before ICI initiation	Cancer-related (82%), cancer-unrelated (9%), immune-related (9%)
Arbour (2018) (17)	640	232	65	90 (prednisone)	PD-1/PD-L1 inhibitor	Orally/IV, ≥10 mg; within 30 days pre- or post-ICI	Cancer-related (68%), other (32%)
Drakaki (2020) (20)	862	466	69 (61–76)	501	PD-1/PD-L1 inhibitor	Within 14 days pre-ICI and 1–30 days post-ICI	Not available
At ICI initiation							
Adachi (2020) (16)	296	206	70 (64–76)	30	Nivolumab	At ICI initiation	Cancer-related (77%), cancer-unrelated (13%), other (10%)
Frost (2021) (23)	153	90	69 (40–86)	37 (prednisolone)	Pembrolizumab	55 ± 33 mg; at ICI initiation	Brain metastases (5%), COPD (20%), immune-related (27%), other (48%)
Hendriks (2019) (25)	1,025	646	64 (30–93)	141	PD-1/PD-L1 inhibitor	At ICI initiation	Brain metastases (100%)
Post-ICI administration							
de Giglio (2020) (19)	413	273	63 (30–92)	49	PD-1/PD-L1 inhibitor	Orally/IV, 40 mg (5–225 mg); during 1–8 weeks post-ICI	Cancer-related (78%), immune-related (12%), other (10%)
Fucà (2019) (24)	151	89	<65 (42%) / ≥65 (58%)	35	PD-1/PD-L1 inhibitor; CTLA-4 inhibitor + PD-1/PD-L1 inhibitor	280 mg (20–875 mg); within 1–28 days post-ICI	Immune-related (11%), supportive care (54%), other (35%)
Lauko (2021) (26)	171	75	64	36	PD-1/PD-L1 inhibitor; CTLA-4 inhibitor ± PD-1/PD-L1 inhibitor	27 mg (5–107 mg); within 1–30 days post-ICI	Brain metastases (100%)
Ricciuti (2019) (27)	650	310	66 (25–92)	93 (prednisone)	CTLA-4 inhibitor + PD-1/PD-L1 inhibitor	Orally/IV, ≥10 mg; within 1–24 h post-ICI	Cancer-related (71%), cancer-unrelated (29%)
Scott (2018) (28)	236	78	68 (62–74)	66 (prednisone)	Nivolumab	Orally/IV, ≥10 mg (10–180 mg); within 1–30 days post-ICI	Cancer-related (66%), immune-related (17%), other (17%)
Other/mixed timing or indication-specific							
Dumenil (2018) (21)	67	46	<70 (58%) / ≥70 (42%)	10	Nivolumab	Orally/IV, 25 mg (12.5–37.5 mg); during first ICI cycle	Brain metastases (100%)
Yamaguchi (2020) (30)	131	98	77 (75–87)	Not available	Pembrolizumab/Nivolumab	Not available	Immune-related adverse events (100%)

ICI, Immune Checkpoint Inhibitor; PD-1, Programmed Death Receptor-1; PD-L1, Programmed Death Ligand-1; CTLA-4, Cytotoxic T-Lymphocyte-Associated Protein 4; IV, Intravenous; COPD, Chronic Obstructive Pulmonary Disease.

### 3.3 Quality assessment results

Quality assessment was conducted using the Newcastle-Ottawa Scale, which evaluated each study based on selection, comparability, and outcome criteria. The total scores of the included cohort studies ranged from 7 to 9, with the majority achieving a score of 9, indicative of high methodological quality. A few studies scored 7 or 8 due to minor limitations in specific domains, such as the ascertainment of exposure or the adequacy of follow-up. Overall, the studies were deemed to be of moderate to high quality, thereby supporting the robustness and reliability of the meta-analysis findings (Table 2).

**Table 2:** The quality assessment according to Newcastle-Ottawa Scale of each cohort study.

Study	Selection			Comparability		Outcome			Total score
	Representativeness of the exposed cohort	Selection of the non -exposed cohort	Ascertainment of exposure	Demonstration that outcome of interest was not present at start of study	Comparability of cohorts on the basis of the design or analysis	Assessment of outcome	Was follow-up long enough	Adequacy of follow up of cohorts	
Adachi (16)	1	1	1	1	2	1	1	1	9
Arbour (17)	1	1	1	1	2	1	1	1	9
Cortellini (18)	1	1		1	1	1	1	1	7
de Giglio (19)	1	1		1	1	1	1	1	7
Drakaki (20)	1	1	1	1	2	1	1	1	9
Dumenil (21)	1	1		1	1	1	1	1	7
Facchinetti (22)	1		1	1	1	1	1	1	7
Frost (23)	1	1	1	1	1	1	1	1	8
Fucà (24)	1	1	1	1	2	1	1	1	9
Hendriks (25)		1	1	1	2	1	1	1	8
Lauko (26)	1	1	1	1	2	1		1	8
Ricciuti (27)	1	1	1	1	2	1	1	1	9
Scott (28)	1	1	1	1	2	1	1	1	9
Taniguchi (29)	1	1	1	1	2	1		1	8
Yamaguchi (30)	1	1	1	1	2	1	1	1	9

### 3.4 Impact of glucocorticoid administration on progression-free survival in patients receiving immune checkpoint inhibitors

**Figure 2:**
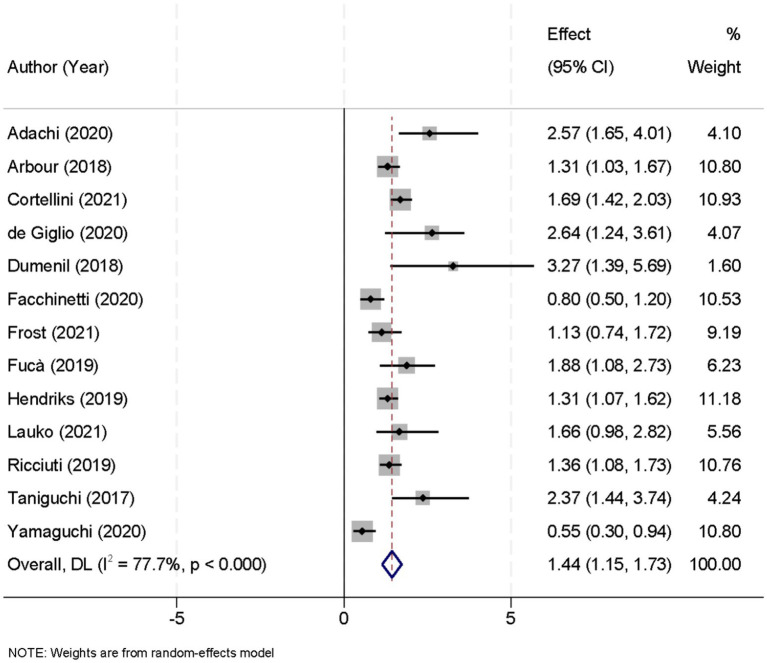
Forest plot showing the effect of glucocorticoid administration on progression-free survival (PFS) in patients treated with immune checkpoint inhibitors (ICIs).

**Figure 3:**
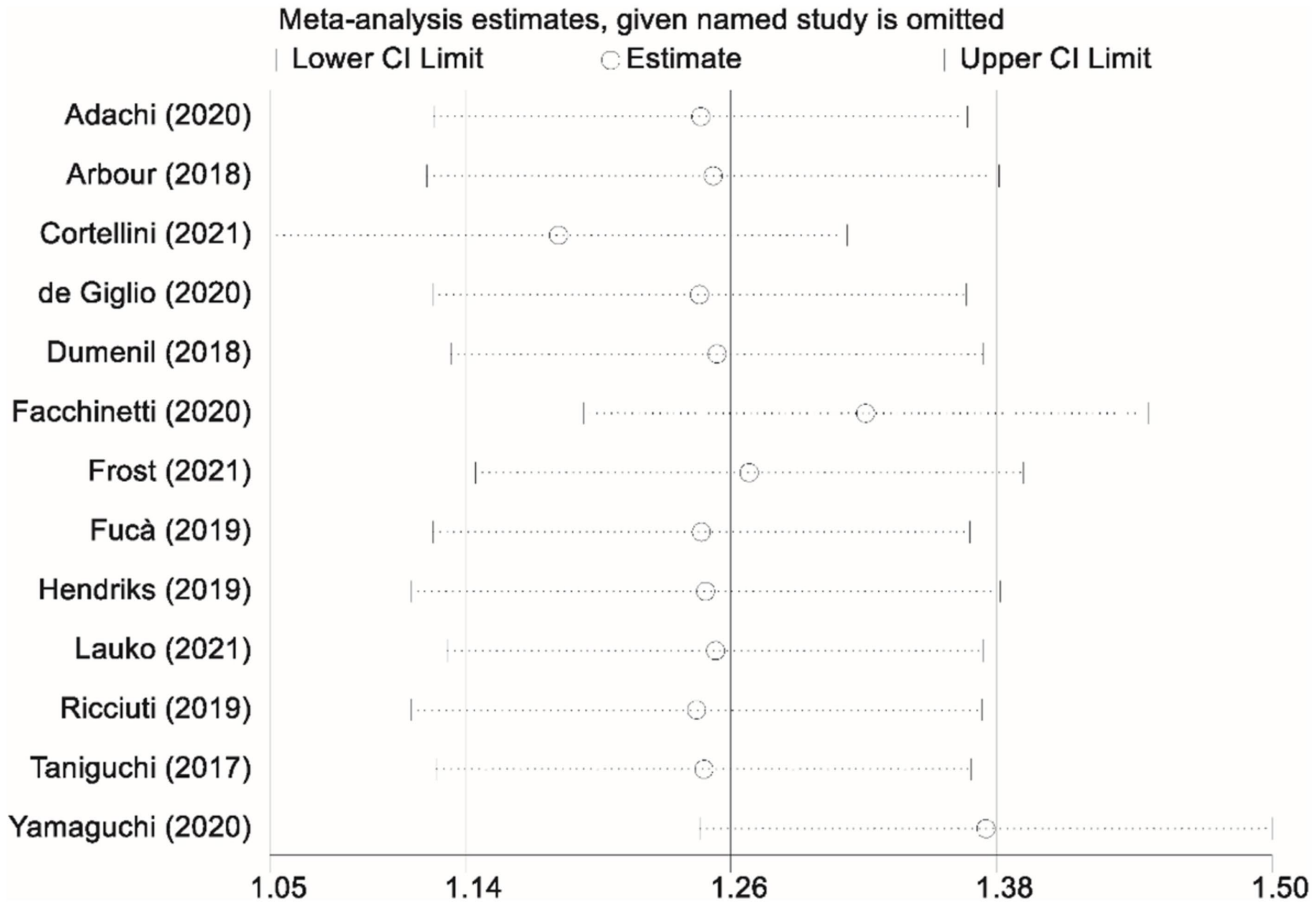
Sensitivity analysis assessing the impact of glucocorticoid administration on progression-free survival (PFS) in patients receiving immune checkpoint inhibitors (ICIs).

Thirteen studies contributed data on progression-free survival (PFS) comparing patients who received glucocorticoids with those who did not. Owing to significant heterogeneity among these studies (I^2^ = 77.7%, *p* < 0.001), a random-effects model was applied to pool the results. The combined analysis yielded a pooled HR of 1.44 (95% CI, 1.15–1.73; *p* < 0.001), indicating that glucocorticoid use was associated with a significantly increased risk of disease progression relative to non-use (Figure 2). To ensure the robustness of this finding, a sensitivity analysis was performed by sequentially excluding each study. This leave-one-out analysis confirmed that the overall PFS result remained stable and robust, demonstrating that the pooled effect estimate was not unduly influenced by any single study (Figure 3).

### 3.5 Impact of glucocorticoid administration on overall survival in patients receiving immune checkpoint inhibitors

**Figure 4:**
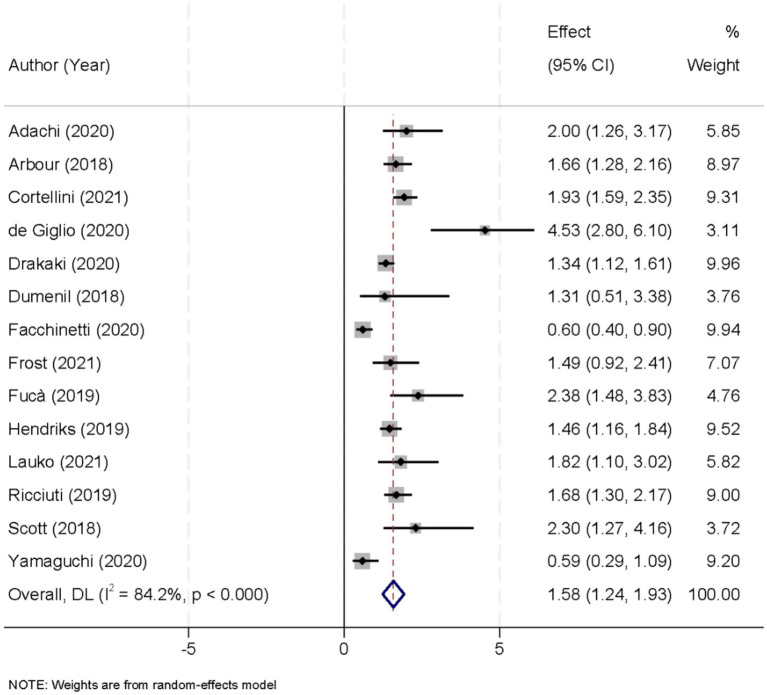
Forest plot displaying the effect of glucocorticoid administration on overall survival (OS) in patients treated with immune checkpoint inhibitors (ICIs).

**Figure 5:**
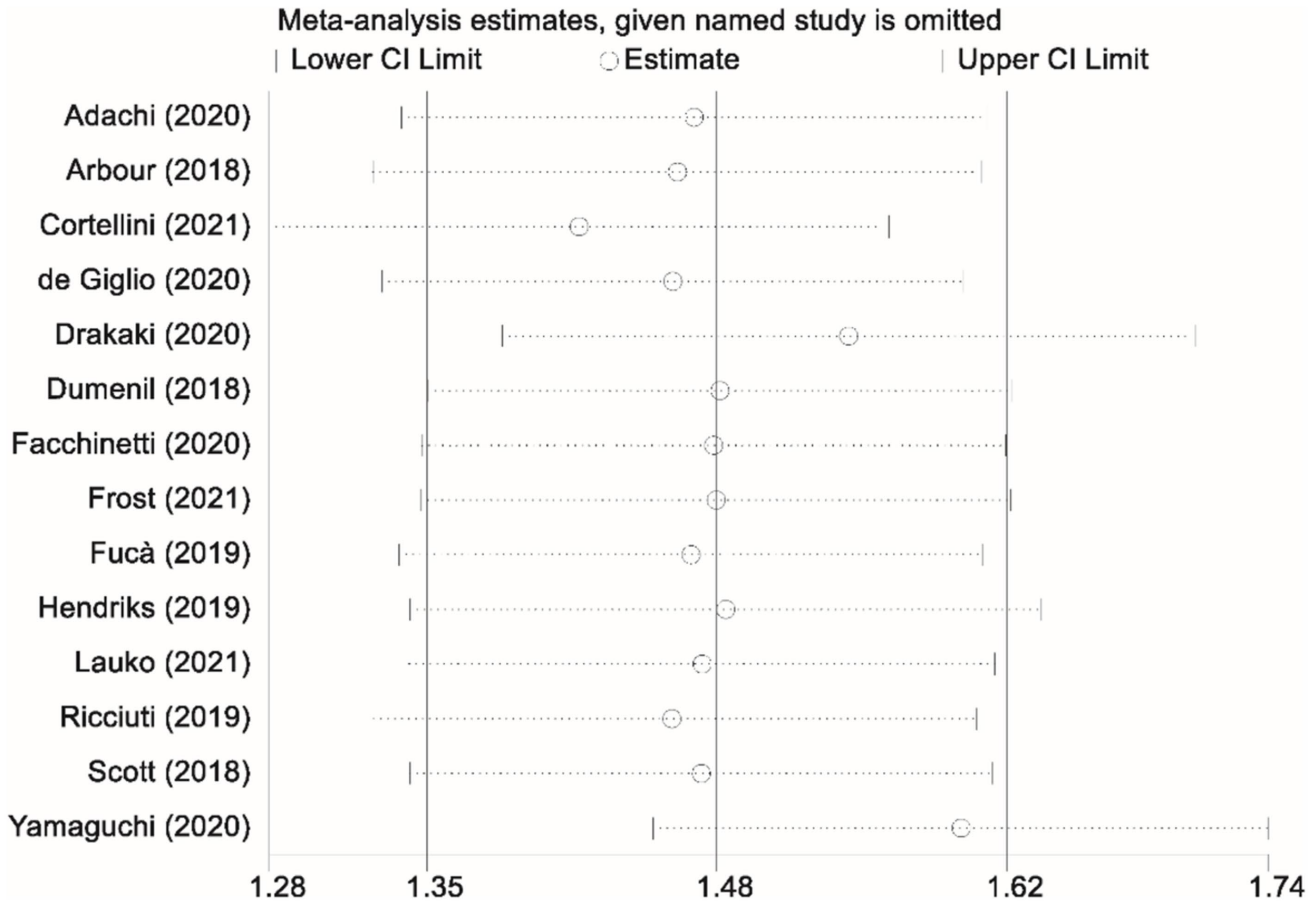
Sensitivity analysis evaluating the impact of glucocorticoid administration on overall survival (OS) in patients receiving immune checkpoint inhibitors (ICIs).

Fourteen studies were included in the evaluation of overall survival (OS) outcomes based on glucocorticoid exposure. Due to considerable heterogeneity across these studies (I^2^ = 84.2%, *p* < 0.001), a random-effects model was employed to synthesize the data. The pooled analysis revealed a HR of 1.58 (95% CI, 1.24–1.93; *p* < 0.001), signifying those patients receiving glucocorticoids experienced a markedly higher risk of mortality compared to those who did not (Figure 4). A sensitivity analysis, conducted by sequentially removing each study from the analysis, confirmed the stability of the overall OS estimate. This rigorous evaluation demonstrated that the observed association between glucocorticoid use and reduced OS was consistently robust despite the exclusion of any individual study (Figure 5).

### 3.6 Subgroup analysis of glucocorticoid administration timing on survival outcomes

A subgroup analysis was performed to evaluate the impact of glucocorticoid administration timing on survival outcomes in NSCLC patients treated with ICIs (Table 3). In the subgroup where glucocorticoids were administered before ICI initiation, five studies were included, showing pooled HRs of 1.21 (95% CI, 0.85–2.01; *p* = 0.15) for PFS and 1.31 (95% CI, 0.69–2.28; *p* = 0.39) for OS, indicating no significant association with adverse survival. By contrast, three studies assessing glucocorticoid administration at ICI initiation demonstrated significant associations with poorer outcomes, with pooled HRs of 1.38 (95% CI, 1.08–2.15; *p* < 0.001) for PFS and 1.56 (95% CI, 1.15–2.01; *p* < 0.001) for OS. Furthermore, in the subgroup where glucocorticoids were administered after ICI initiation, five studies consistently reported significantly worse outcomes, with pooled HRs of 1.98 (95% CI, 1.51–2.62; *p* < 0.001) for PFS and 2.28 (95% CI, 1.61–3.41; *p* < 0.001) for OS. Collectively, these findings suggest that glucocorticoid use, particularly when administered at or after ICI initiation, is significantly associated with an increased risk of disease progression and mortality, whereas pre-ICI use does not appear to exert a statistically significant impact on survival.

**Table 3:** Subgroup analysis of the impact of glucocorticoid administration timing on PFS and OS.

Glucocorticoid administration timing	PFS				OS			
	No. of studies	HR (95% CI)	*P*-value	Effect model	No. of studies	HR (95% CI)	*P*-value	Effect model
Before ICI initiation	5	1.21 (0.85, 2.01)	0.15	Random-effects	5	1.31 (0.69, 2.28)	0.39	Random-effects
At ICI initiation	3	1.38 (1.08, 2.15)	<0.001	Random-effects	3	1.56 (1.15, 2.01)	<0.001	Random-effects
After ICI initiation	5	1.98 (1.51, 2.62)	<0.001	Random-effects	5	2.28 (1.61, 3.41)	<0.001	Random-effects

ICI, Immune Checkpoint Inhibitor; HR, Hazard Ratio; CI, Confidence Interval; PFS, Progression-Free Survival; OS, Overall Survival.

In addition to the leave-one-out sensitivity analyses, we further conducted subgroup-based exclusion analyses to assess the robustness of the pooled results. Specifically, we sequentially excluded all studies categorized as pre-ICI use, at-ICI initiation use, or post-ICI use. The exclusion of each subgroup did not materially alter the overall direction of the association between glucocorticoid exposure and survival outcomes. For progression-free survival, pooled HRs remained within the range of 1.39–1.51 across exclusion scenarios, and for overall survival, pooled HRs ranged from 1.52–1.63. Although minor variations in effect size were observed, the associations consistently indicated that glucocorticoid use was linked to poorer outcomes. These findings reinforce that no single subgroup of studies disproportionately influenced the overall effect estimates, thereby supporting the robustness of our conclusions.

### 3.7 Meta-regression results

Feasibility thresholds were met for timing of exposure and, for a subset of studies, ICI class; other moderators (analytic method, indication, dose category) were insufficiently reported across studies to support stable modeling. In timing-based models (reference: pre-ICI exposure), post-ICI corticosteroid use showed a directionally positive association with both PFS and OS (larger HRs), consistent with our subgroup findings; however, coefficients did not reach statistical significance after Knapp–Hartung adjustment, and the reduction in between-study variance was modest. Models incorporating ICI class did not materially alter pooled effects and explained little heterogeneity. Due to sparse and heterogeneous reporting, meta-regression by analytic approach, clinical indication, or dose category was underpowered and therefore not undertaken in the main analysis. Overall, exploratory meta-regression did not identify a robust study-level moderator that fully accounted for the observed heterogeneity.

### 3.8 Assessment of publication bias

**Figure 6:**
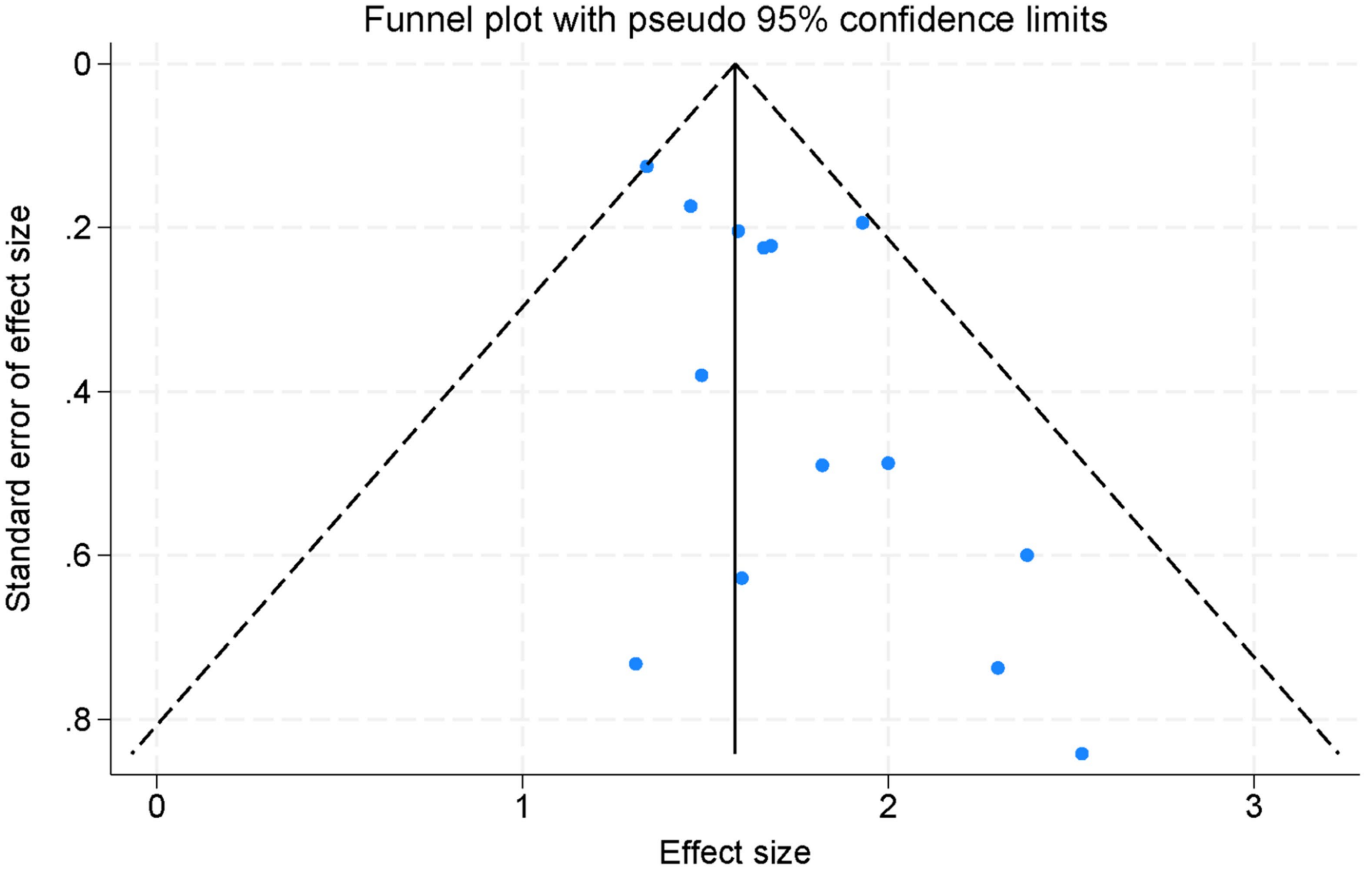
Funnel plot assessing publication bias across all studies included in the meta-analysis.

Visual inspection of the plots revealed a symmetric distribution of effect sizes, indicating that no significant publication bias was detected (Figure 6). Consistently, Egger’s regression test did not demonstrate significant small-study effects for either PFS (*p* = 0.42) or OS (*p* = 0.37), further suggesting that publication bias was unlikely. Although funnel plots and Egger’s test have inherent limitations when the number of studies is moderate, the current findings indicate that publication bias is unlikely to have substantially influenced the results of this meta-analysis.

### 3.9 GRADE assessment of evidence quality

**Figure 7:**
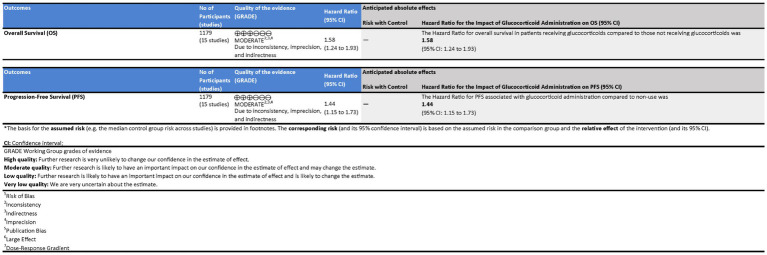
Assessment of the quality of evidence in patients receiving immune checkpoint inhibitors (ICIs) with glucocorticoid administration.

Using the GRADE approach, we systematically evaluated the certainty of evidence for key outcomes in the impact of glucocorticoid administration on OS and PFS in patients receiving ICIs (Figure 7).

The evidence for OS was assessed as Moderate due to significant concerns regarding inconsistency and imprecision despite a large effect and low risk of bias. The HR for OS in patients receiving glucocorticoids compared to those who did not was 1.58 (95% CI: 1.24 to 1.93), indicating a higher risk of mortality associated with glucocorticoid use. Although statistically significant, the broad confidence intervals reflect considerable uncertainty, necessitating cautious interpretation.

For PFS, the quality of evidence was also rated as Moderate, influenced by inconsistency across studies and imprecision in effect estimates. However, the evidence showed a clear large effect with no publication bias. The Hazard Ratio (HR) for PFS associated with glucocorticoid administration compared to non-use was 1.44 (95% CI: 1.15 to 1.73), suggesting that glucocorticoid use was significantly associated with an increased risk of disease progression.

Both outcomes were deemed to have Moderate quality evidence, with the effect sizes providing statistically significant findings. However, the broad confidence intervals and moderate heterogeneity reduce the certainty of the estimates, urging caution in the interpretation of these results.

## 4 Discussion

ICIs have transformed the therapeutic landscape of NSCLC by reactivating the host immune system to target tumor cells. Agents targeting PD-1/PD-L1 and CTLA-4 pathways have demonstrated significant improvements in overall survival and durable responses in advanced NSCLC. However, the effectiveness of ICIs can be affected by concomitant medications, notably glucocorticoids, which are commonly used in this patient population (31, 32). Glucocorticoids are administered for various clinical indications, including the management of cancer-related symptoms, mitigation of immune-related adverse events, and treatment of comorbid conditions. Their inherent immunosuppressive properties, however, raise concerns about a potential reduction in ICIs efficacy (33). This meta-analysis evaluated the association between glucocorticoid use and survival outcomes in NSCLC patients receiving ICI therapy. Our pooled analyses, comprising 13 studies for PFS and 14 for OS, demonstrated that glucocorticoid administration is significantly associated with poorer outcomes. Specifically, glucocorticoid use was linked to a pooled HR for PFS of 1.44 (95% CI, 1.15–1.73; *p* < 0.001) and a pooled HR for OS of 1.58 (95% CI, 1.24–1.93; *p* < 0.001), indicating increased risks of disease progression and mortality. Although we aimed to investigate the dose–response relationship, a formal analysis was precluded by substantial heterogeneity in dosage reporting across studies. Nevertheless, prior evidence suggests that higher steroid doses, particularly those administered for severe irAEs, may be associated with worse clinical outcomes, which is consistent with the overall trends observed in our analysis (9–11).

The findings from our analysis are biologically plausible given the immunosuppressive nature of glucocorticoids. ICIs exert their antitumor effects by reinvigorating cytotoxic T-cell responses, whereas glucocorticoids broadly suppress immune function, including T-cell proliferation, cytokine production, and antigen presentation (34, 35). This mechanistic antagonism may underlie the observed association between glucocorticoid use and diminished ICI efficacy. Furthermore, sensitivity analyses demonstrated consistent results for both PFS and OS, suggesting that our pooled estimates are robust and not driven by any single study. Subgroup analysis revealed that the timing of glucocorticoid administration plays a critical role in modulating ICI outcomes (36, 37). Glucocorticoid use after ICI initiation was significantly associated with worse PFS (HR = 1.98, 95% CI: 1.51–2.62) and OS (HR = 2.28, 95% CI: 1.61–3.41), while pre-ICI administration showed no significant impact on survival. These findings suggest that glucocorticoid exposure during the early immune activation phase of ICI therapy may disrupt the antitumor immune cascade (38). Clinical strategies to minimize immunosuppressive interventions during this critical period, particularly high-dose or prolonged corticosteroid use, may be warranted to preserve therapeutic efficacy.

The heterogeneity of the NSCLC population should also be considered when interpreting our findings. Disease stage, including the presence of brain metastases or other disseminated disease, can significantly influence both prognosis and treatment decisions. Several included studies specifically involved patients with brain metastases, such as Dumenil et al. (21), Hendriks et al. (25), and Lauko et al. (26), in which corticosteroids were administered to manage neurological symptoms or cerebral edema. In such contexts, glucocorticoids may have a palliative benefit and their use may reflect more advanced disease status rather than being a direct modifier of ICI efficacy. This introduces a potential indication bias, whereby poorer outcomes associated with corticosteroid use may be confounded by the underlying disease severity. Moreover, our subgroup analysis clearly demonstrated that glucocorticoid administration after the initiation of ICIs, rather than prior to it, was significantly associated with worse progression-free and overall survival. This temporal distinction suggests that glucocorticoid use may interfere with the antitumor immune activation phase, particularly when used early in the ICI treatment course. Nonetheless, the non-significant impact of pre-ICI glucocorticoid use on outcomes may be due to either lower doses, different indications (e.g., symptom control), or reduced overlap with the therapeutic window of ICI-induced immune activation. Finally, while our meta-analysis robustly pooled hazard ratios for both OS and PFS, we acknowledge that staging information (e.g., TNM classification or metastatic burden) was incompletely reported across studies, precluding formal subgroup analyses based on disease stage. Future meta-analyses incorporating individual patient-level data (IPD) would allow for more granular exploration of these clinically relevant subpopulations.

Emerging preclinical and translational evidence supports the biologic rationale that systemic glucocorticoids may impair the efficacy of ICIs by disrupting antitumor immune responses (39). Glucocorticoids inhibit T-cell proliferation, induce apoptosis, suppress dendritic cell function, and downregulate key cytokines (e.g., IFN-*γ*, IL-2), all of which are crucial for effective ICI-mediated immunity (40). In murine models, glucocorticoids reduce CD8^+^ T-cell infiltration and promote an immunosuppressive tumor microenvironment. Translational data in NSCLC have similarly shown that early or concurrent glucocorticoid use correlates with diminished peripheral T-cell activation and suboptimal radiographic responses (41). These effects may be most detrimental when steroids are administered at ICI initiation, a critical period for T-cell priming. Taken together, these mechanistic insights provide a biologically plausible explanation for the observed associations between glucocorticoid exposure and reduced ICI efficacy.

Although previous studies have consistently shown that the development of irAEs is associated with improved prognosis in NSCLC patients receiving ICIs, our findings indicate a worse survival outcome among patients who received glucocorticoids during ICI therapy (42, 43). This apparent contradiction can be reconciled by considering the severity of irAEs and the intensity of corticosteroid use. Notably, the study by Shimomura et al. (44) demonstrated that while irAEs alone were associated with better outcomes, the administration of high-dose corticosteroids within 60 days, particularly in response to severe irAEs such as pneumonitis—was significantly correlated with worse overall survival. In contrast, patients who received low-dose corticosteroids for milder irAEs did not exhibit a significant survival disadvantage compared to those not receiving corticosteroids. These findings suggest that it is not irAEs per se, but rather the necessity for high-dose immunosuppression, that may compromise the efficacy of ICIs. In our meta-analysis, many included studies lacked detailed stratification by irAE severity or corticosteroid dose, limiting our ability to fully disentangle this relationship. Future studies with patient-level data are warranted to clarify the prognostic impact of steroid dosing and timing in the context of irAE management.

This meta-analysis has several strengths. First, it is the most up-to-date and comprehensive synthesis to date evaluating the impact of glucocorticoid use on ICI efficacy specifically in NSCLC, incorporating a large pooled sample from diverse clinical settings. Second, the use of a prespecified protocol based on the PRISMA framework and a rigorous quality assessment via the Newcastle-Ottawa Scale enhances methodological transparency and reliability. Third, stratified analyses by timing of glucocorticoid administration provide novel insights into temporal effects on survival, which may inform clinical decision-making. Additionally, sensitivity analyses and GRADE evaluation further strengthen the robustness and interpretability of our findings. These methodological advantages collectively increase the credibility and clinical relevance of our results. Several limitations of this meta-analysis should be acknowledged. First, the majority of included studies were retrospective in nature, which makes the findings susceptible to selection bias, residual confounding, and selective reporting. Second, some studies were single-center cohorts, which may limit the generalizability of our results to broader NSCLC populations. Third, heterogeneity in glucocorticoid administration, including timing, dosing, and clinical indication, complicates causal interpretation, particularly given the limited reporting of dosing details and the severity of irAEs across studies. Fourth, the non-significant association observed for pre-ICI steroid use may reflect limited statistical power and clinical heterogeneity rather than a true absence of effect, warranting cautious interpretation. Fifth, the potential influence of unmeasured confounders such as tumor burden, performance status, and concomitant therapies cannot be excluded. Finally, although both funnel plot inspection and Egger’s regression test did not indicate significant small-study effects, the ability of these methods to detect publication bias is limited when the number of studies is moderate. Moreover, because the evidence base is derived predominantly from retrospective studies, selective reporting and publication bias cannot be completely excluded. Future studies should prioritize prospective, multicenter designs with standardized reporting of glucocorticoid timing, dosage, and indications, as well as systematic collection of patient-level data on disease burden, performance status, and treatment context. Such approaches will be critical to disentangling causal effects from confounding and to clarifying the clinical scenarios in which glucocorticoid use most significantly compromises the efficacy of ICIs.

## 5 Conclusion

In NSCLC patients receiving ICI therapy, glucocorticoid use might be associated with poorer outcomes, particularly when administered after ICI initiation. In contrast, pre-ICI glucocorticoid use may not significantly affect patient prognosis. These findings suggest that the timing of glucocorticoid administration could influence ICI effectiveness and underscore the need for cautious glucocorticoid management during immunotherapy.

## Data Availability

The raw data supporting the conclusions of this article will be made available by the authors, without undue reservation.
